# A multi-scale approach for percolation transition and its application to cement setting

**DOI:** 10.1038/s41598-018-33918-6

**Published:** 2018-10-25

**Authors:** Achutha Prabhu, Jean-Christophe Gimel, Andrés Ayuela, Silvia Arrese-Igor, Juan J. Gaitero, Jorge S. Dolado

**Affiliations:** 1 División de Construcción Sostenible, TECNALIA, Parque Tecnológico de Bizkaia, Astondo Bidea, Edificio 700, 48160 Derio, Spain; 2 MINT, UNIV Angers, INSERM 1066, CNRS 6021, Université Bretagne Loire, IBS-CHU, 4 rue Larrey, 49933 Angers, France; 3 Centro de Física de Materiales, Centro Mixto CSIC-UPV/EHU, Paseo Manuel Lardizabal 5, 20018 San Sebastián, Spain; 4 Donostia International Physics Center, Paseo Manuel Lardizabal 3, 20018 San Sebastián, Spain; 5 Faculty of Civil Engineering and Geosciences, Delft University of Technology, Stevinweg 1, 2628 CN Delft, The Netherlands; 6 MATCON, Associated Unit CSIC-TECNALIA, Parque Tecnológico de Bizkaia, Astondo Bidea, Edificio 700, 48160 Derio, Spain

## Abstract

Shortly after mixing cement grains with water, a cementitious fluid paste is formed that immediately transforms into a solid form by a phenomenon known as setting. Setting actually corresponds to the percolation of emergent network structures consisting of dissolving cement grains glued together by nanoscale hydration products, mainly calcium-silicate-hydrates. As happens in many percolation phenomena problems, the theoretical identification of the percolation threshold (i.e. the cement setting) is still challenging, since the length scale where percolation becomes apparent (typically the length of the cement grains, microns) is many times larger than the nanoscale hydrates forming the growing spanning network. Up to now, the long-lasting gap of knowledge on the establishment of a seamless handshake between both scales has been an unsurmountable obstacle for the development of a predictive theory of setting. Herein we present a true multi-scale model which concurrently provides information at the scale of cement grains (microns) and at the scale of the nano-hydrates that emerge during cement hydration. A key feature of the model is the recognition of cement setting as an off-lattice bond percolation process between cement grains. Inasmuch as this is so, the macroscopic probability of forming bonds between cement grains can be statistically analysed in smaller local observation windows containing fewer cement grains, where the nucleation and growth of the nano-hydrates can be explicitly described using a kinetic Monte Carlo Nucleation and Growth model. The most striking result of the model is the finding that only a few links (~12%) between cement grains are needed to reach setting. This directly unveils the importance of explicitly including nano-texture on the description of setting and explains why so low amount of nano-hydrates is needed for forming a spanning network. From the simulations, it becomes evident that this low amount is least affected by processing variables like the water-to-cement ratio and the presence of large quantities of nonreactive fillers. These counter-intuitive predictions were verified by ex-professo experiments that we have carried out to check the validity of our model.

## Introduction

Networks are in general a group of interconnected entities following certain characteristics and produce complex structures such as the brain, the internet etc.^1^. In many cases, networks emerge and expand over time by following simple processes that can be described using a given set of rules^2^. These local rules can be inter related and may contribute to varying sets of properties. Nucleation and growth (N&G) processes can be considered as one of such rules and is observed in many naturally occurring processes such as crystal growth^3^, self assembly^4^, etc. It can be explained as the formation, organization and aggregation of simpler units constituting a geometrical growth scheme, leading to the formation of large intricate structures like crystals, films, fractals, etc. Even though N&G process are best studied locally where each local events can be monitored, the complex nature of the resulting systems makes it difficult to estimate all the resultant emergent properties from this scale. Percolation is such an emergent feature of network systems. It is a well-studied critical phenomenon and in its simplest form, describes the formation and growth of clusters of simpler entities (building blocks - for example: particles) or bonds leading to the formation of a spanning cluster or network structure and is characterized by a threshold. Processes involving network growths leading to percolation have great practical interest and are commonly used to describe many real-world systems. However, identification and extraction of percolation threshold is a challenging task at local scales, since the length scale where percolation becomes apparent is many times larger than the basic building blocks. Simulation studies offer approximate results, but the finite size effects usually play a big role and restrict their scope^5^. In the present study, a statistical approach is presented to identify the percolation threshold from a local scale in the context of cement setting, employing a multi-scale cement hydration model.

The use of cementitious materials dates back to the age of Romans. Easily available raw materials, flexibility of use, durability, price and competent mechanical properties makes them the most used construction materials of the present world in terms of volume. The high demand and increased production of cement however results in an increased carbon footprint, amounting to 5% of total anthropogenic carbon dioxide emission in the world^6^ and about 1.7% of total global water withdrawal^7^. As a result, designing cheap “green cements” having lower carbon footprint, lower water withdrawal and improved properties has become a priority in the construction sector. Despite the historic usage and continued research, understanding how their functional properties vary with the involved physicochemical processes remains a challenge, because of its complexity.

**Figure 1:**
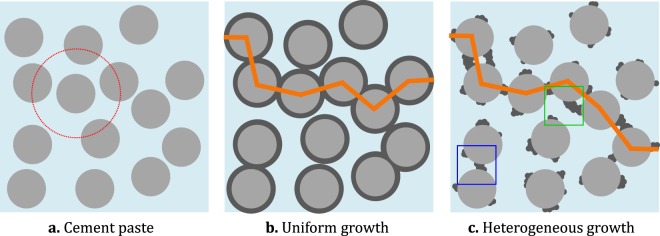
(**a**) Schematic representation of the cement paste, where light grey cement particles are dispersed in light blue water. The dotted circle represents the neighbourhood of the selected central grain. (**b**) and (**c**) Show the formation of a percolating cluster (shown by orange line segments connecting grain centres) formed by the precipitated C-S-H (in dark grey) linking the grains. (**b**) Depicts the uniform growth of C-S-H around the grains, as represented by the HCSS model and (**c**) shows a heterogeneous growth of C-S-H. The local details at a smaller length scales (represented by coloured squares in (**c**)) are required to correctly describe bond formation.

When cement powder is mixed with water, a viscous fluid (cement paste) is formed. The properties of this granular nonhomogeneous media evolve over time as the hydration products, chiefly the calcium-silicate-hydrate (C-S-H) gel, continue to form. Nanoscale C-S-H precipitate and grow onto the partially dissolved micron sized cement grains, gluing them together in the process^8^. As this process continues, evolving local networks consisting of cement grains held together by C-S-H phase are formed which eventually leads to the formation of a percolating network (see Fig. 1), at which point the cement paste is said to have set^9^. Setting is accompanied by a transition of the liquid cement paste to a solid form along with an increase in resistance to applied stress^10^. The time taken for this percolation transition i.e., setting time and properties at this point, like the degree of hydration at setting (*α*_set_) are important engineering tools for characterization of cement formulations, because early age properties point to the properties of the final hardened cement^11^. Understanding the formation and structure evolution of hydration products is therefore the key to control properties of hardened cement paste and eventually concrete.

Cement paste is a multi-scale porous material composed of unhydrated cement particles and small hydrated crystallites embedded into the amorphous nanostructured C-S-H gel^12–14^. While C-S-H is known to have a layered structure akin to tobermorite minerals at the molecular level^10,15–18^, neutron and X-ray scattering^11,15^, microscopy^19^ and molecular simulation^20^ studies indicate that it precipitates as roughly spherical particles of around 5 nm diameter, that aggregate and evolve into a colloidal gel like structure^8,21,22^. The properties of these nano C-S-H aggregates evolve over time, and are shown to be responsible for the properties of bulk hydrated cement^23–25^.

C-S-H phase formation in the inter cement grain spaces is kinetically driven and is identified to a N&G process^10,21,26^. Early attempts to describe C-S-H formation include analytical Avrami type growth models^27,28^, where a constant density C-S-H phase grows uniformly around many point nuclei distributed in the inter-grain space. Growing C-S-H phase from different nuclei eventually impinge, interpenetrate and fill the space. A better description of C-S-H formation is known to be given by the so called Boundary Nucleation and Growth (BNG) models, where point nucleation takes place only on the surfaces of cement grains. Proposed by Cahn^29^ to describe transformations in polycrystalline materials, the BNG model was applied to cements for the first time by Thomas^30^, and afterwards elaborated by Scherer *et al*.^31^ and Scherer^32^ to account for both possible anisotropic and confined growths. Under the BNG set of models, C-S-H phase grows around point surface nuclei in an Avramian fashion and leads to nonhomogeneous growth fronts around cement grains^32^. Even though simple, these analytical models are capable of reproducing the heat rate evolution for cement hydration to a large extent^30^ by varying the nucleation and growth rates. However, they largely overestimate the amount of C-S-H formed along the hydration time. For instance, if the volume fraction occupied by C-S-H at a given time *t* is given by *X*(*t*), the values predicted by the Avrami model and the BNG models at the peak in the d*X*/d*t* function are ${X}_{{\rm{peak}}}\sim 0.5$ and ${X}_{{\rm{peak}}}\sim 0.25$ respectively^32^. These figures largely differ from the experimental value of $\sim$0.071^33^. This clearly demonstrates the inefficiencies of these models to capture the essential features of C-S-H formation.

Since N&G models ignore the dissolution of cement grains, one might think that more sophisticated computational models which couple the cement dissolution kinetics to microstructure formation via rate equations of chemical reactions and mass balance considerations between cement grain phases and hydration products^34–38^ are required. While this is true at large time scales where cement grains are noticeably shrunk because of the hydration process, at early ages the degree of hydration is so low (e.g. ∼0.071 at heat rate peak^33^ and ∼0.04 at setting^33^) that cement dissolution can hardly justify why the BNG models predict hydration degrees that grossly exceed the experimental values. A more plausible explanation to their failure was already addressed by Bishnoi and Scrivener^38^, pointing to the presence of a certain texture in the hydrates that evolves and densifies over time. In this scenario, nano-colloidal models where the C-S-H phase is described as an aggregate of nanoscale colloidal building blocks^8,21,22,39,40^ have gained acceptance to explain such texture densification process. The packing variation of nanoscale C-S-H has been shown to reproduce results from nanoindentation experiments, while the long lasting granular dynamics of C-S-H particles is suggested to cause creep^40^. Recently a colloidal N&G model was proposed by Gonzalez-Teresa *et al*.^21^ to explain the early formation of C-S-H. In spite of assuming a bulk nucleation (i.e. an Avramian style of nucleation), this colloidal N&G model was sufficiently able to capture the growth of C-S-H and its evolving texture on an equal footing, and accounted simultaneously for the calorimetry behaviour and hydration degree results.

The Hard Core Soft Shell (HCSS) model^32,41^ is considered as the basic model to study cement setting. It considers precipitation of thin constant density soft interpentrable C-S-H shells around hard uniform spherical cement grains suspended in water, thereby including provisions to account for the effects of liquid to solid weight ratio (*L*/*S*), an important processing parameter in cement field. As more layers gets added, C-S-H from neighbouring grains overlap and link them to create increasingly large clusters, eventually forming a percolating cluster (schematic shown in Fig. 1b). However, HCSS simulations on setting is shown to overestimate the *α*_set_, especially at higher *L*/*S*^42^.

Despite numerous analytical and simulation studies, linking setting of bulk cement paste to the nanoscale C-S-H growth and morphology remains an open question^14^. Setting becomes apparent at the bulk scale, a length scale many orders of magnitude larger compared to the nanoscale C-S-H building blocks. This presents limitations in designing simulations and leads two mutually exclusive scenarios: (**i**) at larger length scales, it becomes increasingly impractical to accommodate nanoscale details, and (**ii**) at nano scale, setting cannot be directly identified. In addition, simulating differences in local reactivity leading to varying local structures increases the computational complexity.

In the present study, we report on computational simulations employing a statistical approach to evaluate cement setting which enables for the first time a multi-scale cement hydration model including the nanoscale details. The model takes into account different bulk mix conditions by varying *L*/*S*, amount of substituent material etc., and the C-S-H precipitation was explicitly modelled at the nanoscale, where evolving C-S-H morphologies were observed. To this end, the Avramian-like colloidal N&G model proposed in^21^ has been adapted to BNG-like version, where the nucleation can occur on the cement grain surfaces. We find that the *α*_set_ predictions made by the model is consistent with our ex-professo experiments using ordinary cement paste and cement paste formulations in which a part of cement has been replaced with sand, demonstrating the importance of nanoscale C-S-H morphology on setting. The model provides insights into the nano-structural evolution of hydration products which in turn can enhance our knowledge to design new smart cements, additives^43,44^ and supplementary cementitious materials^45^, with specific properties and lower carbon emissions. Furthermore, the approach presented can be employed to study other evolving network systems. While structural evolution in the local scale provides insights into the system at hand, a judicious comparison of the obtained results with known bulk phenomena can predict emerging features including percolation, which cannot be detected at smaller length scales.

## Results and Discussion

### The model

**Figure 2:**
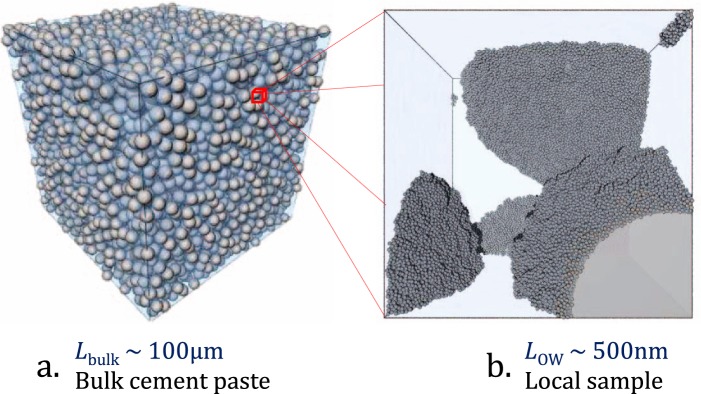
The simulation scheme showing (**a**) the bulk cement paste with cement grains represented as grey monodisperse hard spheres suspended in semitransparent light blue water and (**b**) a randomly chosen local sample showing C-S-H growth in dark grey.

Two length scales were employed in our model (see Fig. 2). Large cubic Random Hard Sphere (RHS) systems (edge length *L*_bulk_), assuming the cement grains to be spheres with diameter *d*, and the rest water, were used to represent the bulk cement paste systems such that its properties like *L*/*S* are determined by the occupation fraction (*ϕ*) of the spheres (see methods section for details). Numerous randomly selected smaller regions (cubic observation windows with edge length *L*_OW_) of this bulk system constituted the local samples and consisted of smaller cubic boxes with exposed cement grain surfaces and water filled inter-grain spaces. Nanoscale C-S-H precipitation was simulated in these local samples following a simple nucleation and hierarchical growth scheme adapted from González-Teresa *et al*.^21^. This gives a detailed picture of the C-S-H structural evolution in the local observation windows and the obtained results were averaged to get the emerging bulk property. However, the local simulations are incapable of directly identifying setting.

For large cement paste samples (*L*_bulk_ ≫ *d*), C-S-H precipitation in a small region ($\sim$*d*) is independent on what occurs in the rest of the system. Likewise, a link formation between neighboring cement grains due to the precipitated C-S-H can be considered independent on any other link formation in the system. Under these conditions, the process of link formation leading to setting becomes analogous to off-lattice uncorrelated bond percolation problem^46^, where cement grains act as the sites bonded by the C-S-H phase growing from their surfaces. For this case, the probability to obtain a percolated system depends only on the relative amount of established bonds and there exists a threshold. The average probability 〈*p*〉 for the connectivity transition has been calculated by randomly distributing bonds between grains within the exploration range *d* to (1 + *ε*) ⋅ *d*, where *ε* is a dimensionless variable, and checking for percolation. This method has been found to be prone to finite size effects when the system size is not large enough. Hence, the values of 〈*p*〉 have been extrapolated to infinite *L*_bulk_ in order to evaluate the critical bond percolation probability (*P*_c_)^46,47^ (see supplementary information).

For a given *L*/*S*, a wide range of *P*_c_ values can be obtained by varying *ε*^46^. Hence, the choice of *ε* is important in order to compare percolation and setting. While very small *ε* may not include the neighbouring grains, very large values may show the unrealistic link formation with distant grains, rendering both choices meaningless. The first coordination shell was selected as the ideal value of *ε* considering the early age at which setting takes place and because of the direct accessibility to the grains in this range. For RHS systems, this selection correspond to first minima in their pair correlation function and also satisfies the condition ε < *d* ≤ *L*_OW_. Interestingly, for generally employed *L*/*S* ranging from 0.3 to 0.5, this exploration range returns in average ∼ 12 neighbors within the first coordination shell and a *P*_c_∼0.12 (see supplementary information). This means that 12% of formed links out of all possible links within the said range leads to percolation. The low *P*_c_ value evidences that the nature and morphology of C-S-H growth can play a major role on setting by influencing linkage kinetics.

**Figure 3:**
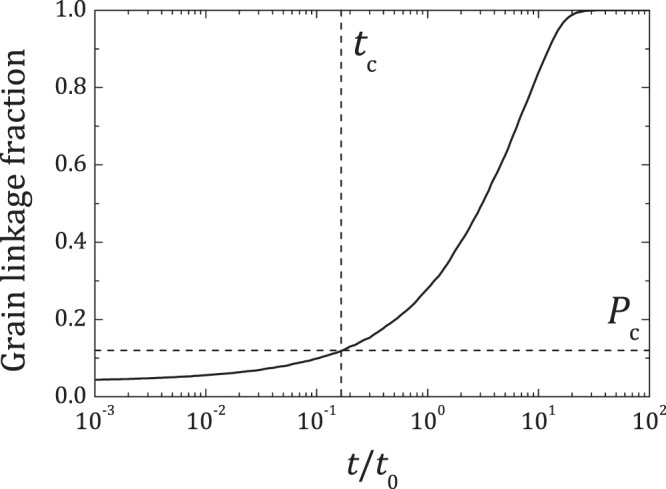
Grain linkage fraction plotted as a function of time (*t*/*t*_0_) for *L*/*S* = 0.32 and *L*_OW_ = *d* for 1000 trials. The horizontal dashed line corresponds to the critical percolation threshold, *P*_c_, and the vertical dashed line shows corresponding setting time *t*_c_/*t*_0_.

Local samples help to explicitly monitor C-S-H growth leading to linkage with neighbouring grains. However, our choice *L*_OW_ = *d*, is not large enough to enclose the entire first coordination shell of an average grain. Even if *L*_OW_ were to be larger, the selection of local system was not centred around grains, limiting the possibility of extracting a local measurement on the links formed by an average grain. As an alternative, link formations in local systems were related to setting/percolation in the parent system. During simulations, formation of new grain links by the growing C-S-H was monitored at each time step. For a large sample set, good statistics on these grain linkage times was obtained. Assuming all possible links are formed at large times, the fraction of grain links formed (grain linkage fraction, GLF) was plotted as a function of time (Fig. 3 (left)). A sigmoid is obtained, that may not necessarily start from zero, because the initial placement of C-S-H particles themselves may form links with neighbouring grains, especially for lower *L*/*S*.

Comparing GLF cosrresponding to *P*_c_ gives the setting time *t*_c_. The obtained *t*_c_ is virtually free from finite size effects, since there is no direct involvement of the macro scale cement paste. The degree of hydration at setting (*α*_set_) was then calculated from the knowledge of the amount of precipitated C-S-H (*ϕ*_C−S−H_) employing the law of mass conservation (see supplementary information). It is worth mentioning that employing *L*_OW_ ≥ *d*, the same criterion given by the coarseness approach, finite size effects on *ϕ*_C−S−H_ and GLF were found to be negligible at early ages, when setting takes place (see supplementary material). This method gives access to macroscopic system properties at the meso-scale virtually free from finite size effects, exposing its multi-scale nature.

### Degree of hydration at setting (*α*_set_) for normal cement pastes

**Figure 4:**
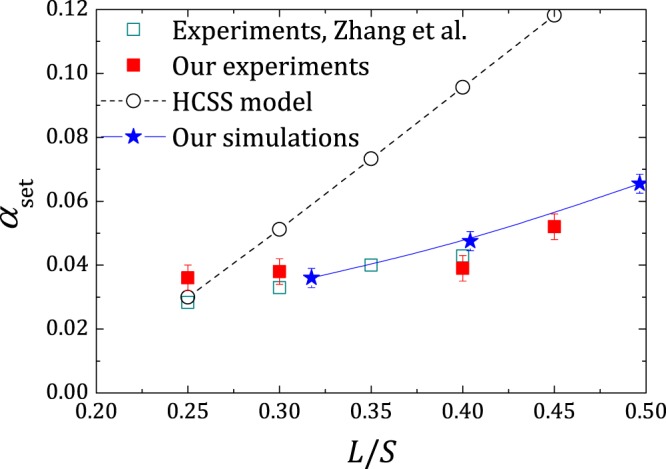
Degree of hydration at setting (*α*_set_) as a function of *L*/*S*. For our simulations, *L*_OW_ = *d* was employed. Data for HCSS model (*ρ*_C−S−H_ = 2.0 g cm^-3^) and experimental data by Zhang et al. adapted from ref.^33^. Curves are guides to the eyes. Error bars in our simulation data correspond to 2% error on the determination of *t*_c_/*t*_0_. Error bars in our experimental data correspond to 95% confidence intervals.

Employing the presented multi-scale model, normal cement pastes with varying *L*/*S* were simulated. From the obtained GLF curves, *t*_c_ was noted and subsequently *α*_set_ was calculated for each case. Figure 4 compares the *α*_set_ values predicted by our simulations and our select experiments with the HCSS model and previously reported experimental results (data adapted from refs^33,42^). It is clear that our multi-scale nano-colloidal model correctly recovers the experimental results for *α*_set_. The increasing discrepancy in *α*_set_ for larger *L*/*S* observed in the HCSS model has been attributed to agglomeration effects, which were omitted in the original model^48^. However, we find that the observed low *α*_set_ values can be explained on the basis of heterogeneous C-S-H growth, average distance between the grains and fraction of grain links at setting.

**Figure 5:**
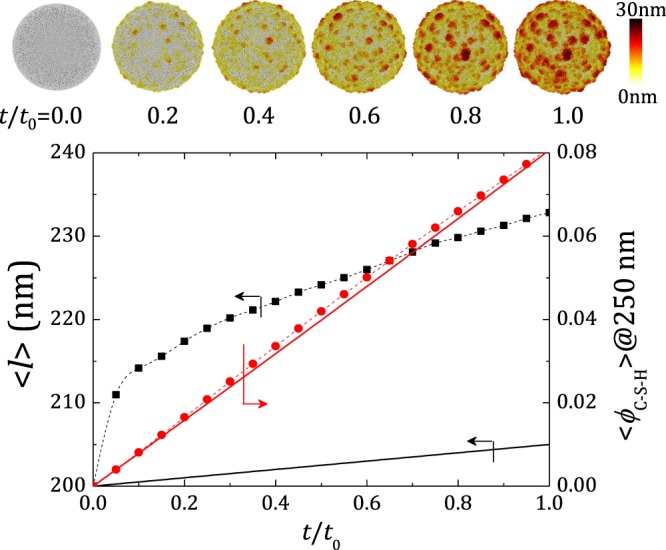
Top: Typical snapshots showing early age evolution of growth morphology on an isolated cement grain with at various times. Colours indicate height profile from the grain surface. Bottom: The average distance to the farthest C-S-H particle, $\langle l\rangle$ (in black), and volume fraction of C-S-H, *ϕ*_C−S−H_ (in red), obtained in a virtual spherical shell around the grain at 250 nm plotted as a function of time. Symbols connected by dashed curves represent our model and solid lines represent the HCSS model. *ϕ*_C−S−H_ of our model is corrected for initial nuclei.

For a better understanding about how the heterogeneous C-S-H growth affects *α*_set_, the employed C-S-H N&G scheme was simulated on an isolated cement grain (averaged over 100 trials, see snapshots in Fig. 5). For comparison, HCSS growth with C-S-H density *ρ*_C−S−H_ = 2.0 g cm^-3^ and radius increment rate 5 nm (*t*/*t*_0_)^−1^ was also simulated on an isolated grain. The two growth models were characterized by calculating the C-S-H volume fraction (*ϕ*_C−S−H_) in an imaginary spherical shell around the grain. For facility, the volume corresponding to the initial nuclei was subtracted for the colloidal model. It was observed that both models show similar trend in terms of *ϕ*_C−S−H_ (See Fig. 5 (bottom)). However, the average distance to the farthest C-S-H particle and the distance covered by the HCSS model (both calculated from the grain centre) showed a drastic difference. While HCSS model followed a linear behaviour, the nano-colloidal model showed a sudden initial increase, which later slowed down. This difference originates from the irregular C-S-H precipitation leading to rougher growth fronts, and is clearly visible in the snapshots.

In the RHS cement paste system, the average inter-grain distance increases with increasing *L*/*S*. Following the uniform growth approach put forward by the HCSS model, this means that more C-S-H is expected to form at setting as *L*/*S* increases. However, the experimental results does not show a considerable increase in *α*_set_, and the discrepancy with HCSS model increases at higher *L*/*S* (see Fig. 4). The evolution of the average distance to the farthest C-S-H particle at early ages demonstrates that our heterogeneous growth model has a higher reach and hence an increased grain linkage probability, at the expense of comparatively lower amount of C-S-H (see Fig. 5 (bottom)). In other words, the uniform growth front in HCSS model produces an excess amount of C-S-H to cover a given distance or in turn to form a grain link, compared to our model. For the *L*/*S* range under consideration, the average surface-to-surface distance to the nearest-neighbour grain is of the order of C-S-H particle size and does not show a large variation within the range. Furthermore, uncorrelated bond percolation simulations in RHS systems show that in average about 2 bonds per particle is sufficient to obtain a percolating network (see supplementary information). In effect, a few well located heterogeneous C-S-H growths on grain surfaces are sufficient to form links with the closest neighbours and to lead to setting with consistently low *α*_set_ values, exposing the importance of nanoscale morphology of precipitated hydration products. This topological picture matches well with the microstructure revealed by Zingg *et al*.^49^ from cryo-SEM analysis.

Figure 4 also shows that our ex-professo experiments are consistent with previous observations of Zhang *et al*.^33^. At first, this can be considered intriguing, because the cements employed in the studies are different. In the light of our findings this actually indicates that for regular cements, *α*_set_ is not largely affected by small differences in chemistry of the system, but is determined by the fastest reacting phase in the system, which in this case is the same (alite) for both cements. To verify this finding, complexity of the system was increased by replacing a part of cement with comparatively non reactive sand.

### Degree of hydration at setting (*α*_set_) for substituted cement pastes

Finely divided supplementary cementitious materials and admixtures are often added to cements for controlling properties of fresh and hardened mixes and as well as for economic reasons^44^. These materials, generally known as fillers, affect the formation of hydration products in various ways such as by dilution, changing the system chemistry^50,51^, etc. Our model is capable of handling such cement formulations where a part of cement has been replaced by a filler material. As an example, we simulated a series of cement paste formulations (*L*/*S* = 0.30) where parts of cement have been substituted with comparatively non-reactive sand. For comparison, experiments for the same set were performed in parallel.

In terms of simulation, introduction of substituent material corresponds to randomly selecting the correct fraction of the spheres in the closest parent RHS system and identifying them as the filler material. The difference in filler chemistry was modelled by varying the C-S-H precipitation on the filler grains. Owing to lack of details, simulations were restricted to two limiting cases: (**i**) where the filler is “inactive” and no C-S-H precipitation takes place on filler grains, and (**ii**) where the filler is equally “active” as cement grain in initiating C-S-H precipitation on its surface. The term “activity” in this case refers to the capability of inducing C-S-H formation on the respective grain surface. The chemically inert nature of sand implies that it cannot impart chemical effects, and the hydration product comes solely from the reaction of cement grains.

Experiments show that initial setting time increases with increase in sand substitution (see supplementary information). This points to the fact that the introduction of external materials such as sand can change the kinetics of hydration. This delay in setting has a complex rationale originating from the lower amount of cement (dilution), difference in relative surface area, the difference in interactions (chemical or physical) with the substituent material, etc., or any of their combination^44^. In simulations, a delay in *t*_c_ was observed when C-S-H precipitation was absent on the sand grains, causing C-S-H on the cement grains to grow for longer times and larger extents to form the links. *t*_c_ for the “active” case remained unchanged, since it was identical to the initial model for normal cements in terms of C-S-H growth. It must be noted that in the case of substituted cement pastes, the sand grains also contribute to the formation of percolating network by participating in the link formation.

**Figure 6:**
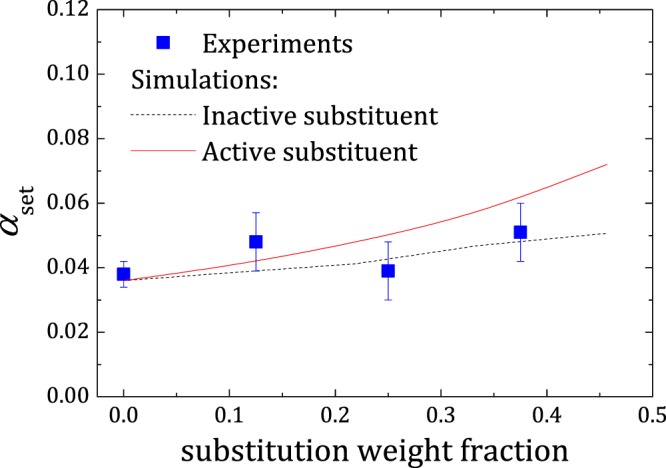
Experimental results on degree of hydration at setting, (*α*_set_) as a function of substitution fraction for sand substituted cements with *L*/*S* = 0.30 compared with simulations (*L*_OW_ = *d*), where nucleation occurs on the cement grains (dotted curve) and on all grains (solid curve). Error bars in simulation data omitted for clarity. Error bars in experimental data correspond to 95% confidence intervals.

Figure 6 shows *α*_set_ predictions by simulations compared to the experimental results for various sand substitution fraction. The experimental data is found to lie within the prediction window formed by the two limiting cases, further validating our model. A recent study demonstrated that C-S-H precipitation on sand grains at early ages is comparable to that on cement grains^50^, thus suggesting “active” filler (case **ii**) to be closer to reality. Simulating C-S-H precipitation on both grains in equal amounts results in systems identical to the initial model of normal cement, apart from the fact that sand is chemically inert in this case. The evaluated *α*_set_ values for this case showed a small relative increase with increasing filler substitution, while the inactive filler (case **i**) remained largely insensitive. This observation mainly comes from the additional surface area available with increasing filler quantity, requiring more cement to be converted to C-S-H at setting. According to percolation theory, a minimum amount of grain links needs to be formed in order to reach setting, and this is independent on the system chemistry. Given the fastest reacting phase is the same, there exists a minimum *ϕ*_C−S−H_ that needs to be precipitated at setting for all the cases. Furthermore, we find that even with 50% substitution, the difference in *α*_set_ between the two limiting cases is exceptionally small. Given the fact that sand is inactive in case **i**, this observation strengthens our previous finding that the system chemistry has little effect on *α*_set_ and that it is determined by the fastest reacting phase.

Recent studies have shown that limestone, another widely used filler material, produces slightly higher C-S-H growth compared to sand by influencing the system chemistry^50,51^. Limestone is found to chemically react and contributes to a faster C-S-H precipitation on its surface, thereby further altering the hydration kinetics. Apart from chemically interacting fillers, hydration kinetics can also be altered by introducing very small quantities of chemical additives like calcium chloride, which is known to exhibit an accelerating effect^52^. Our simulations suggest that even though the hydration kinetics and setting time can be altered by various means, large differences in *α*_set_ values are not expected. Formation of grain links is the core of setting process and remains unchanged irrespective of the filler employed, indicating that under general usage conditions *α*_set_ is essentially a critical quantity. Zhang *et al*.^33^ have experimentally determined the setting times and *α*_set_ for various additives and also probed the effect of temperature. Even though the setting times were found to be dependent on the nature and amount of additive and temperature, *α*_set_ values were nearly identical in all cases, further proving the correctness of our reasoning.

## Conclusions

In short, a multi-scale model based on uncorrelated bond percolation transition was presented. Using the statistical approach employing kinetically evolving spatially uncorrelated local samples, the process leading to bond formation was studied in detail to smaller length scales. While averaging the local samples gives the bulk property, percolation was identified by comparing the local bond formation kinetics to threshold of appropriate bond percolation model. This method proves useful for various percolation process, where the existing models fail to provide details to the smaller length scales. As an example, the approach was applied to cement hydration. The degree of hydration at setting (*α*_set_) for normal cements with various *L*/*S* cases predicted by our model was well in accordance with the experiments. Using a combination of simulations, experiments and previously reported data, we conclude that even though the chemistry and composition of the system may vary the cement hydration kinetics, it is the topology that determines the average grain linkage at setting. Hence, given the fastest reacting phase remains the same, *α*_set_ is largely unaffected for the generally employed *L*/*S*, even with large amounts of non-reactive filler material.

In the future, a time dependent model can be developed by varying characteristic nucleation time (see ref.^21^), while the growth model can be modified to include different growth modes or directional growths to identify different hydration products. This can be further improved by including different characteristic growth intervals to represent effects of composition and chemical additives which can alter growth morphology and the final property of the system^53^. Introducing additional nucleation sites in the inter-grain spaces mimics seeding, where C-S-H particles or other suitable chemicals are added to cement paste to accelerate the hydration process^52^. Further improvements can include a slight polydispersity^54^ to C-S-H particles. By fine tuning these modifications, the system can be brought closer to the reality and eventually, real life setting times can be predicted.

Besides the studies on cement setting, our model is also extensible to simulate other cases. For systems constituting evolving networks, the concept of connecting local events to events in a larger perspective as shown in this work, provides a new pathway to study the underlying local processes with great detail. This can lead to prediction of emerging features including percolation, which cannot be detected at smaller length scales, opening up new pathways to explore phenomena like creep^40^.

## Methods

### Multiscale model

Our multi-scale approach takes into account the micro-scale structure of the cement paste and the nano-scale morphology of the precipitated C-S-H. Since a direct incorporation of nano-scale details to the micro-scale is computationally challenging, they were modelled in two steps. First a sufficiently large micro-scale cement paste system was generated. Later, parts of this micro-system were randomly sampled using a smaller observation window of appropriate size. Nano-colloidal C-S-H precipitation was modelled within these local systems with great detail to the morphology. Averaging a large number of such local systems gave the properties of the parent micro-system. A detailed description of the model is given below:

#### Micro-scale model

Micro-scale cement paste was modelled using cubic boxes (with edge *L*_bulk_) of Random Hard Sphere (RHS) systems with a given total occupation fraction (*ϕ*^0^) and the rest was assumed to be filled with water (Fig. 2a). Depending on simulation conditions, a fraction ${\varphi }_{{\rm{g}}}^{0}$ of the hard spheres was considered as cement grains and the rest ${\varphi }_{{\rm{s}}}^{0}={\varphi }^{0}-{\varphi }_{{\rm{g}}}^{0}$ as the substituent (in this case sand) grains. This was achieved by randomly selecting a given number of grains corresponding to ${\varphi }_{{\rm{s}}}^{0}$ and setting them as the substituent. By varying *ϕ*^0^ and ${\varphi }_{{\rm{g}}}^{0}$ of the RHS systems, cement pastes with various liquid to solid weight ratio (*L*/*S*) were generated, having known system properties such as inter grain distance distribution^41,55^, a key topological parameter in our model (see supplementary information). For simplicity, the grains (cement and substituent) were considered to be monodisperse, with a diameter (*d*) equal to the characteristic size of the most populated fraction of real cements (400 nm). This can be justified, because the particle size in normal cements generally follow a Rosin-Rammler distribution and is strongly dominated by the total number of the smaller fraction^10^ (see supplementary information, wherein the particle size distribution of the cement employed in our experiments is given). Besides, it is worth remembering that at sufficiently high packing densities, polydispersity has little effect on the mean surface-surface distance between neighbour particles, [page 176 of reference^41^]. Further, Scherer *et al*.^42^ have already shown using the HCSS model that polydispersity has little effect on *α*_set_.

#### Scaling down to nano-scale: the hand-shake procedure

Implementing the nano-colloidal model in micron sized boxes becomes increasingly forbidding even for small *L*_bulk_, because the number of C-S-H particles to be modelled becomes too large. This limitation was overcome by simulating C-S-H precipitation in numerous randomly selected smaller regions of the cement paste, followed by averaging the results (See Fig. 2b). A detailed description of this sampling-averaging method is given by Torquato, [page 257 of reference^41^], where a parameter “Coarseness” was introduced to quantify the deviation of the averaged local observable from the bulk property, like porosity. “Coarseness” is dependent on the size and shape of the observation window and tend to stabilize as the size of the observation window increases. In essence, given the size of observation window is large enough, ie. when the “Coarseness” is close to its asymptotic value, the resultant average local property can be considered to be a good representation of the bulk property.

In our simulations, the observation window was modelled as a small cubic box with width edge length *L*_OW_ = 0.25*d*, 0.5*d*, 1*d*, etc. In order to find the optimal *L*_OW_ to be used in the final simulations, a study was carried out to estimate the average local *L*/*S* of the bulk systems under consideration. This involved sampling bulk system, dividing the local system into smaller voxels and calculating the volume occupied by the cement grains by counting the voxel occupancy. It was found that *L*_OW_ ≥ *d* correctly represents the system property: the *L*/*S* of the parent cement paste (see supplementary information). Following this observation, the data presented in this study, unless specified, were obtained from simulations with *L*_OW_ = *d* by averaging at least 1000 local systems.

Breaking the bulk system to numerous local systems bring down the number of C-S-H particles to be simulated to accessible levels (*N* ∼ 10^6^) and thousands of such smaller systems can be simulated in a reasonable time. Assuming there exist no long range correlation in C-S-H precipitation, growth morphology can be successfully studied in the local systems. This further falls in our favour, since it is known that C-S-H precipitation kinetics is found to differ close to the substituent grains and these smaller details can be successfully incorporated and visualized in the local smaller systems, while the collective property gives correct bulk values. In the simulations, it is the volume fraction of the precipitated C-S-H in the available volume that we measure and average from the local systems. The available volume of the water filled space was obtained by the voxel counting method, as explained before.

#### Nano-scale model

Once an appropriate local observation window containing exposed grain surfaces and water filled inter grain space was obtained, the nano-colloidal C-S-H phase formation was simulated. Nucleation, growth and the resulting morphology on hydration products depend on many factors like the interplay of chemical constituents, supersaturation levels^56,57^, temperature, etc. A robust and comprehensive computational framework of precipitation has been recently proposed by Shvab *et al*.^26^, but it turns out to be computationally intensive for its implementation in this study. Here, the educated N&G scheme proposed by Gonzalez-Teresa *et al*.^21^ was employed because it was shown to capture the involved physics at low computational cost. In essence, this model combined a rate-dependent nucleation with a hierarchical growth mechanism that provide different textures by changing two parameters, the final targeted density of nuclei (*η*_0_) and the characteristic nucleation time (*τ*). Here, we employ a site-saturated case (*τ* = 0) and a density of nuclei equivalent to a maximum grain surface coverage *θ* ≈ 0.54. These values are supported by previous analytic models for regular cements^31^ in pure water. However, appropriate values can be used for other types of cements and substituents. C-S-H nuclei were placed with their centres touching the grain surface and their total number (*N*_nuc_) noted. Later, a layer by layer growth mode was adapted around the nuclei, intending to model the proposed auto-catalytic nature of C-S-H growth^52^.

Following ref.^21^, a nucleus was randomly selected at each *t* and a layer of C-S-H particles were placed around it, uniformly distributed in a spherical shell of width 0.5 nm. In the case of an already grown nucleus, growth was attempted on every particle grown from it in the previous steps. No mutual overlap was allowed between C-S-H particles at any time and particle placement outside the box boundaries was not allowed. After each growth event, a virtual linking procedure was carried out, where any two unlinked spheres (cement grains or C-S-H particles) having a surface to surface distance less than 0.5 nm were assumed to be virtually in contact and thus linked. Time (*t*) was then incremented and the simulation continued until the box was filled with C-S-H particles. When *t* reaches *N*_nuc_, all nuclei in the system have been in average entitled to a chance of growth uncorrelated. This was selected as the unit of time (*t*_0_) and the simulation time was expressed in dimensionless unit *t*/*t*_0_. At each time step, *t*/*t*_0_, the C-S-H occupation fraction in the available space (*ϕ*_C−S−H_) and the newly formed grain-(C-S-H)-grain links were noted. A snapshot from such a simulation is shown in Fig. 2b.

### Experimental details

In order to compare the degree of hydration at setting with experimental values, two sets of experiments were conducted. In the first set, cement paste samples were prepared at varying liquid to solid weight ratios (L/S) using an ordinary Portland cement (CEM I-52.5N) and distilled water. In the second set, *L*/*S* = 0.3 was kept constant but cement was partially replaced by finely grounded siliceous sand at different proportions. Particle size distribution of both materials was determined by laser diffraction (Malvern Panalytical Mastersizer 2000) with the objective of verifying that there were not large differences between them (see supplementary information). Setting times were determined using Vicat needle tests according to European standard EN 196-3 but at predetermined *L*/*S* instead of at normal consistency. For the determination of *α*_set_, 100 g of binder were mixed with water at 750 rpm for 1.5 min, followed by 1 min rest and another 1.5 min of mixing at 750 rpm. About 40 mL of the resulting cement paste was poured into a glass vial where it was compacted 60 times in order to remove air bubbles. The vial was then closed and submerged in water at 20 °C for curing. On reaching initial setting time, several paste samples with a volume smaller than 1 mL (about 5 mL in total) were taken from the vials and submerged into liquid nitrogen for more than 15 min to arrest the chemical reaction. In order to minimize the risk of carbonation the top surface of the paste contained on the vial was discarded. Deep-frozen samples from liquid nitrogen were then directly introduced in a lyophilizer (Telstar Cryodos −80) at −77 °C to freeze-dry for 3 days. During the short time elapse between freeze-drying and thermogravimetric measurements, the resultant fine powder was stored in glass containers at vacuum. High Resolution Thermogravimetric measurements (TA Instruments Q500) were run on samples of about 20 mg under 60 mL min^-1^ N_2_ flow as follows: 10 min isotherm at 28 °C; 5 °C min^-1^ heating from 28 °C to 105 °C; 30 min isotherm at 105 °C; and 10 °C min^-1^ high resolution heating from 105 °C to 1000 °C. The degree of hydration at setting, *α*_set_, was determined from the measured weights following refs^58,59^.

## Electronic supplementary material

Supplementary material: A multi-scale approach for percolation transition and its application to cement setting
