# Supplementary material for: A multi-scale approach for percolation transition and its application to cement setting
Source: Sci Rep. 2018 Oct 25;8:15830. doi: 10.1038/s41598-018-33918-6 (PMC6202394; doi:10.1038/s41598-018-33918-6)
Supplement: Supplementary file 1 — Supplementary material: A multi-scale approach for percolation transition and its application to cement setting [file 41598_2018_33918_MOESM1_ESM.pdf]

Supplementary material: A multi-scale approach for percolation transition and its application to cement setting

Achutha Prabhu,[1] Jean-Christophe Gimel,[2] Andrés Ayuela,[3,4]
Silvia Arrese-Igor,[3] Juan José Gaitero,[1,5] and Jorge S. Dolado[3,4,6]

[1] *TECNALIA, División de Construcción Sostenible, Parque Tecnológico de Bizkaia, c/ Geldo, 48160 Derio, Spain.*
[2] *MINT, UNIV Angers, INSERM 1066, CNRS 6021, Université Bretagne Loire, IBS-CHU, 4 rue Larrey, 49933 Angers, France.*
[3] *Centro de Física de Materiales, Centro Mixto CSIC-UPV/EHU, Paseo Manuel Lardizabal 5, 20018 San Sebastián, Spain.*
[4] *Donostia International Physics Center, Paseo Manuel Lardizabal 3, 20018 San Sebastián, Spain.*
[5] *MATCON, Associated Unit CSIC-TECNALIA.*
[6] *Faculty of Civil Engineering and Geosciences, Delft University of Technology, Stevinweg 1, 2628 CN Delft, The Netherlands*

DETAILS ON RELATIONS EMPLOYED

For a Random Hard Sphere (RHS) cement paste system with total volume fraction $\phi^0 = \phi^0_{\mathrm{g}} + \phi^0_{\mathrm{s}}$, where ϕ^0_{g} denotes the initial volume fraction occupied by the cement grains and ϕ^0_{g} the initial volume fraction of the substituent material, the liquid to solid weight ratio (L/S) (or the water to cement weight ratio when the substituent is absent) is given by

$$L/S = \frac{\left(1-\phi^0\right)\cdot\rho_{\mathrm{w}}}{\phi^0_{\mathrm{g}}\cdot\rho_{\mathrm{g}} + \phi^0_{\mathrm{s}}\cdot\rho_{\mathrm{s}}}. \tag{1}$$

Where, ρ_{g}, ρ_{s} and ρ_{w} denote the density of cement grain ($3.15\,\mathrm{g\,cm^{-3}}$), the cement substitute (in the case of sand ρ_{s}=$2.64\,\mathrm{g\,cm^{-3}}$) and water ($1\,\mathrm{g\,cm^{-3}}$), respectively. With the nuclei placed on a grain, the surface coverage (θ) is calculated as

$$\theta = \frac{\pi\cdot r^2_{\text{C-S-H}}\cdot N_{\mathrm{nuc}}}{4\cdot\pi\cdot(d/2)^2}, \tag{2}$$

where $r_{\text{C-S-H}}$ is the radius of a C-S-H particle (here $2.5\,\mathrm{nm}$). For small simulation boxes, we populate the entire grain with nuclei and then consider the nuclei within the box as valid, such that θ remains unaffected. Alternatively, the exposed grain surface area can be estimated used Monte Carlo methods.

As the dissolution precipitation involving C-S-H formation takes place, we denote ϕ_{g} as the grain volume fraction at a given time. Assuming uniform dissolution, the reduction in grain diameter is given by

$$\Delta x = d\cdot\left[1-\left(\frac{\phi_{\mathrm{g}}}{\phi^0_{\mathrm{g}}}\right)^{\frac{1}{3}}\right]. \tag{3}$$

For generally observed degrees of hydration at initial set ($\alpha_{\mathrm{set}}\sim 0.03$), we get $\Delta x\sim 4\,\mathrm{nm}$, smaller than the size of a C-S-H particle. This Δx value is negligible, and our placement of the nuclei being partially overlapping with the grain compensates for this dissolution effect.

At early ages of hydration, the law of mass conservation is valid and dissolution effects are negligible. Thus, we can write

$$\begin{aligned}\left[1-\left(\phi^0_{\mathrm{g}}+\phi^0_{\mathrm{s}}\right)\right]\cdot\rho_{\mathrm{w}} + \phi^0_{\mathrm{g}}\cdot\rho_{\mathrm{g}} + \phi^0_{\mathrm{s}}\cdot\rho_{\mathrm{s}} = \\ \left[1-\left(\phi^0_{\mathrm{g}}+\phi^0_{\mathrm{s}}\right)\right]\cdot\left(1-\phi_{\text{C-S-H}}\right)\cdot\rho_{\mathrm{w}} + \\ \left[1-\left(\phi^0_{\mathrm{g}}+\phi^0_{\mathrm{s}}\right)\right]\cdot\phi_{\text{C-S-H}}\cdot\rho_{\text{C-S-H}} + \phi_{\mathrm{g}}\cdot\rho_{\mathrm{g}} + \phi_{\mathrm{s}}\cdot\rho_{\mathrm{s}}\end{aligned} \tag{4}$$

where $\rho_{\text{C-S-H}}$ is the density of dry colloidal unit ($2.8\,\mathrm{g\,cm^{-3}}$) [1]. For non-reactive sand, no dissolution takes place and $\phi^0_{\mathrm{s}} = \phi_{\mathrm{s}}$. With ϕ_{g} known, we can calculate the degree of hydration (α) as follows

$$\alpha = 1-\frac{\phi_{\mathrm{g}}}{\phi^0_{\mathrm{g}}}. \tag{5}$$

For substituted cements, we choose a reference parent system and set a given amount of randomly selected grains as the substituent particles. This leads to systems that differ in their sand/cement content with relatively unchanged L/S. For the given case, for 0% to 50% substitution by sand in volume (with density $\rho_{\mathrm{s}} = 2.64\,\mathrm{g\,cm^{-3}}$) gives L/S values ranging from 0.32 to 0.34. Our data (given in article) shows that there is no noticeable difference in α_{set} for the experimental data and simulation data in this range.

EVOLUTION OF NUMBER OF NEIGHBORS WITH EXPLORATION RANGE (ε)

The average number of neighbors (N_n) in the exploration range increases with ε (in unit of d) (see figure A1). A meaningful choice to study growth from a central grain should include all directly accessible grains in the close vicinity. This restriction leads us to approximate the first coordination sphere as ε (figure A1). For RHS systems, first coordination sphere corresponds to the first minima in its pair correlation function. This choice gives $N_\mathrm{n} \approx 12$ for our selection of L/S from 0.3 to 0.5.

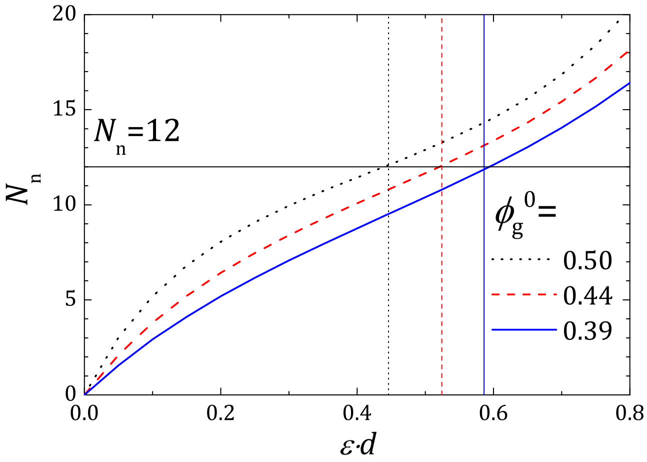

FIG. A1. Evolution of average number of neighbors (N_n) as a function of $\varepsilon \cdot d$ for various ϕ_g^0. The horizontal line marks $N_\mathrm{n} = 12$ and the vertical lines mark the first minima for the corresponding pair correlation function.

ESTIMATION OF UNCORRELATED BOND PERCOLATION THRESHOLD

In order to obtain the uncorrelated bond percolation threshold, simulations were performed on bigger RHS systems ($L = 10d, 20d, 30d, 40d$, etc.). A distance range d to $(1+\varepsilon)\cdot d$ was specified to determine the neighbors, and bonds were distributed between them with a given probability p. When all possible bonds were distributed, the system is tested for the presence of a percolating cluster in at least one direction. After many such realizations, we obtain the fraction of the total systems that has been percolated, P. This procedure was repeated for various p and system sizes (see Figure A2). For this study, the value of ε was chosen such that it is the distance corresponding to the first minimum in the $g(r)$ curve. A minimum of 10000 samples were simulated for calculating P. The value of average bond probability $\langle p\rangle$ and the standard deviation SD= $\left(\langle p^2\rangle - \langle p\rangle^2\right)^{0.5}$ were calculated by integrating over the derivative of P, where

$$\langle p^n\rangle = \int_0^1 p^n \cdot \frac{\mathrm{d}}{\mathrm{d}p} P(p) \cdot \mathrm{d}p \qquad (6)$$

The resultant plot of $\langle p\rangle$ as a function of SD was extrapolated, and the $\langle p\rangle$ value at zero SD is taken as the system size independent percolation threshold, P_c [2, 3] .

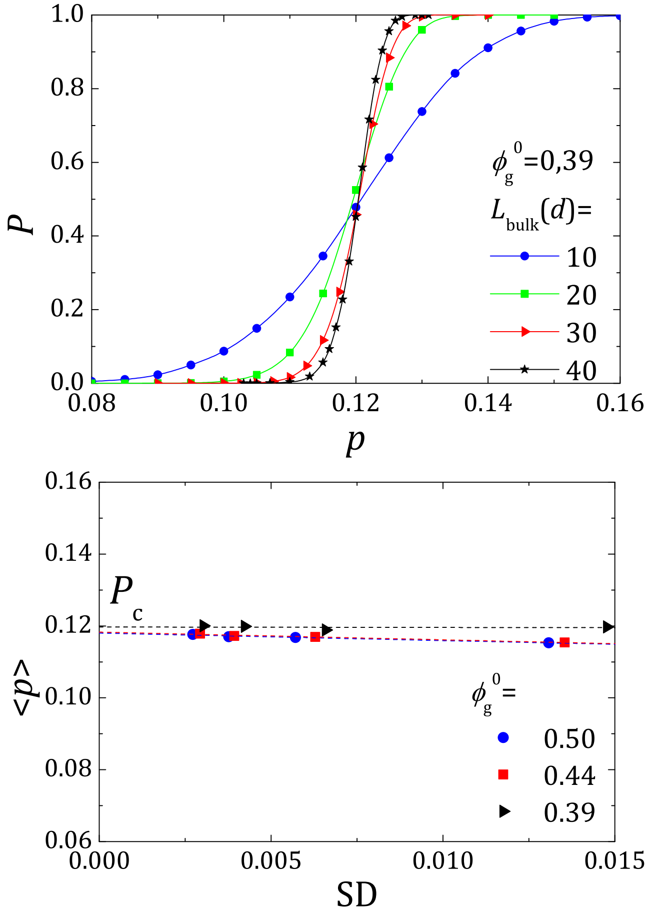

FIG. A2. (top) Fraction of percolated systems P plotted as a function of bond probability p for various L_bulk as shown in the figure. ε was chosen as the distance to the first coordination shell ($\varepsilon \approx 0.585$ for $\phi_\mathrm{g}^0 = 0.39$). Curves are guides to the eyes. (bottom) Extrapolation of $\langle p\rangle$ to zero standard deviation gives P_c.

CHOICE OF L_{OW} BASED ON COARSENESS

The choice of L_{OW} determines the distribution of observed local property or "coarseness" (C), defined as the ratio of standard deviation to the average observed value. The value of C is dependent on the nature of the parent system and the shape and size of the sampling window. For large L_{OW}, an asymptotic limit $C = K \cdot v^{0.5}$ exists, where K is a positive value which depends on L/S and $v = L_{OW}^3$ is the volume of the sampling window [4, p. 261]. The effect of L_{OW} on the evolution of local ϕ_g^0 was studied using a voxel counting method using at least 1000 samples (figure A3). When $L_{OW} \geq d$, the observed C values are close to the corresponding asymptotic values, indicating a good local representation of the parent system.

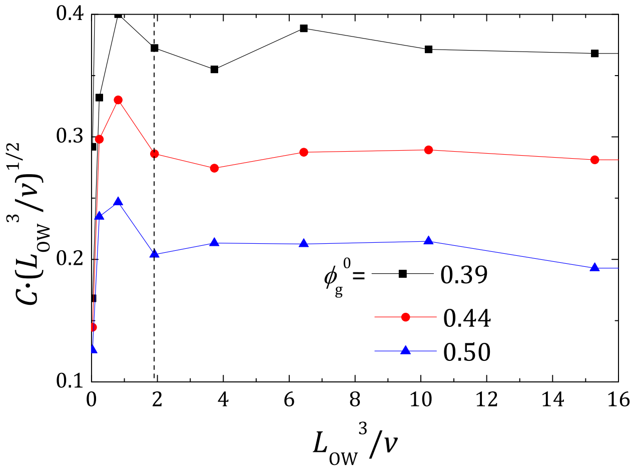

FIG. A3. Scaled coarseness, $C \cdot (L_{OW}/v)^{0.5}$, as a function of the scaled observation volume L_{OW}^3/v for various ϕ_g^0 as indicated. Here $v = \pi/6 \cdot d^3$.

FINITE SIZE EFFECTS ON ϕ_{C-S-H} EVOLUTION

The outward growth of C-S-H continues until it reaches the C-S-H growth from a neighboring grain or the neighboring grain surface itself. Hence the optimal size of the simulation box is determined partially by L/S of the system being modeled. The sampling becomes biased for small box sizes, because samples with large inter grain distance are omitted. The evolution of $\phi_{\text{C-S-H}}$ depends on the available free space, exposed grain surface, and the number of nuclei present in the system. Having $L_{OW} = d$ appears to be enough to avoid large errors for $\phi_{\text{C-S-H}}$ values (see Figure A4) and the grain linkage fraction (see Figure A5). The final value $\phi_{\text{C-S-H}} \sim 0.46$ has been observed previously for the colloidal model. The slight difference in the final values of $\phi_{\text{C-S-H}}$ for small L_{OW} is due to the low number of nuclei leading to relatively lower packing defects.

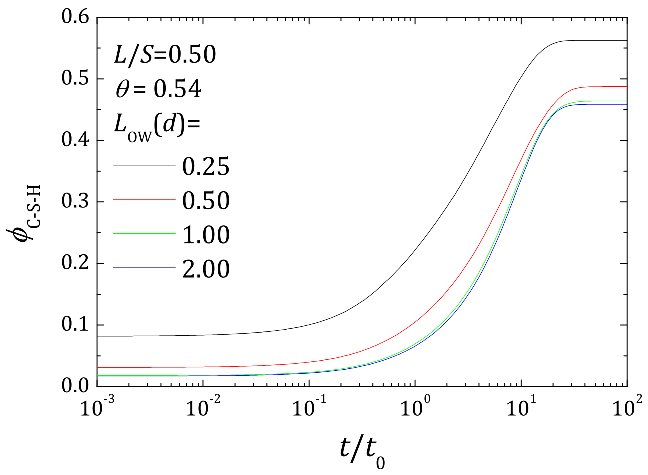

FIG. A4. Evolution of $\phi_{\text{C-S-H}}$ as a function of simulation time for various observation window sizes (L_{OW}) for $L/S = 0.50$ and $\theta = 0.54$.

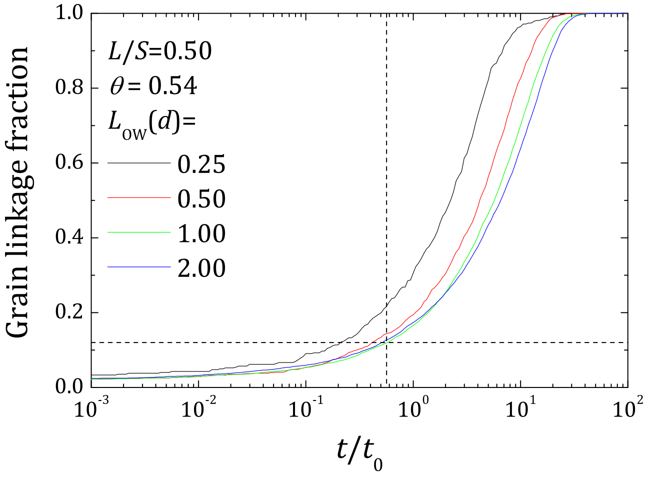

FIG. A5. Grain linkage fraction as a function of time for various system sizes (L_{OW}) for $L/S = 0.50$ and $\theta = 0.54$. The horizontal dashed line corresponds to the uncorrelated bond percolation threshold P_c, and the vertical dashed line indicates corresponding t_c/t_0.

SETTING TIMES OBTAINED FROM VICAT NEEDLE TESTS

Setting times obtained from Vicat needle tests according to European standard EN 196-3 are given in table A1 for plain cement pastes with varying L/S and in table A2 for substituted cements with $L/S = 0.30$ and varying sand substitution fraction.

TABLE A1. Initial setting times for cement pastes with varying L/S.

L/S	0.25	0.30	0.40	0.45
Initial setting time (minutes)	110	140	190	270

TABLE A2. Initial setting times for cement pastes with $L/S = 0.30$ and varying sand substitution fraction.

% substitution (by weight)	0.0	12.5	25.0	37.5	50.0
Initial setting time (minutes)	140	145	170	180	220

PARTICLE SIZE DISTRIBUTION

Particle size distributions were measured for the cement ($\rho_g = 3.15\,g\,cm^{-3}$) and sand ($\rho_s = 2.64\,g\,cm^{-3}$) samples employed in the experiments using laser diffraction, with 2-propanol as the dispersion medium (Figure A6).

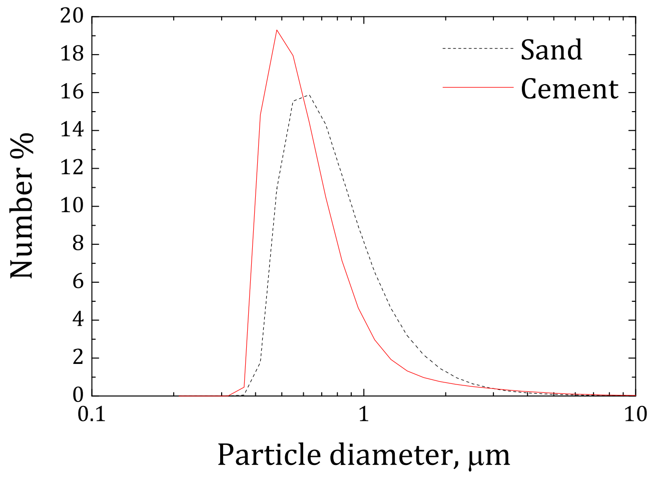

FIG. A6. Obtained particle size distribution for sand and cement.

[1] H. M. Jennings, Cem. Concr. Res. **30**, 101 (2000).

[2] M. Rottereau, J. Gimel, T. Nicolai, and D. Durand, Eur. Phys. J. E **11**, 61 (2003).

[3] J. C. Gimel, T. Nicolai, and D. Durand, J. Phys. A: Math. Gen. **32**, L515 (1999).

[4] S. Torquato, *Random Heterogeneous Materials: Microstructure and Macroscopic Properties*, 1st ed. (Springer-Verlag New York, 2002).
